# Exploring risk factors of pelvic organ prolapse at eastern of Democratic Republic of Congo: a case-control study

**DOI:** 10.1186/s12905-024-03010-5

**Published:** 2024-03-26

**Authors:** Eloge Ilunga-Mbaya, Denis Mukwege, Renaud De Tayrac, Branly Mbunga, Raha Maroyi, Mukanire Ntakwinja, Mushengezi Amani Dieudonné Sengeyi

**Affiliations:** 1 Faculty of Medicine, Department of Gynecology and Obstetrics, University of Kinshasa, Kinshasa, Democratic Republic of Congo; 2 Department of Gynecology and Obstetrics, Université Evangélique en Afrique, Panzi Hospital, Bukavu, Democratic Republic of Congo; 3 Department of Obstetrics and Gynecology, University of Montpellier, Nîmes University Hospital, Montpellier, France; 4 Kinshasa School of Public Health, Faculty of Medicine, University of Kinshasa, Democratic Republic of Congo, Kinshasa, Democratic Republic of Congo

**Keywords:** Low-income countries, Pelvic organ prolapse, Risk factors

## Abstract

**Background:**

Pelvic organ prolapse is a common debilitating condition worldwide. Despite surgical treatment, its recurrence can reach up to 30%. It has multiple risk factors, some of which are particular for a low-resource settings. The identification these factors would help to devise risk models allowing the development of prevention policies. The objective of this study was to explore risk factors for pelvic organ prolapse in a population in eastern Democratic Republic of Congo (DRC).

**Methods:**

This was an unmatched case-control study conducted between January 2021 and January 2022. The sample size was estimated to be a total of 434 women (217 with prolapse as cases and 217 without prolapse as controls). Data comparisons were made using the *Chi-Square* and Student T tests. Binary and multivariate logistic regressions were used to determine associated factors. A *p* < 0.05 was considered significant.

**Results:**

Variables identified as definitive predictors of pelvic organ prolapse included low BMI (aOR 2.991; CI 1.419–6.307; *p* = 0.004), home birth (aOR 6.102; CI 3.526–10.561; *p* < 0.001), family history of POP (aOR 2.085; CI 1.107–3.924; *p* = 0.023), history of birth without an episiotomy (aOR 3.504; CI 2.031–6.048; *p* = 0), height ≤ 150 cm (aOR 5.328; CI 2.942–9.648; *p* < 0.001) and history of giving birth to a macrosomic baby (aOR 1.929; IC 1.121–3.321; *p* = 0.018).

**Conclusions:**

This study identified that Body Mass Index and birth-related factors are definitive predictors of pelvic organ prolapse in a low-resource setting. These factors are potentially modifiable and should be targeted in any future pelvic organ prolapse prevention policy. Additionally, there seems to be a genetic predisposition for prolapse, which warrants further assessment in specifically designed large scale studies.

## Background

The international Continence Society (ICS) defines pelvic organ prolapse (POP) as the descent of the anterior and/or posterior vaginal wall as well as the vaginal apex (uterus or vaginal vault after hysterectomy) [1]. The global prevalence of POP has been recently reported to be around 9% [2]. The overall prevalence of prolapse in the United States is reported to be 21.7% amongst women aged 18 to 83, with rates as high as 27% and 30% in women aged 30 to 49 and 50 to 89 years respectively. The prevalence in low-income countries is estimated to be closer to 20% [2–3]. However, the true prevalence of POP in low-resource settings may be underestimated due to social stigma, shame, and lack of awareness [4].

POP is considered a major cause of morbidity. It impacts negatively on the quality of life and well-being of patients and can represent a significant economic burden for families, particularly with limited healthcare access in low-resource settings. Furthermore, outcomes of surgical management are sometimes suboptimal with recurrence rates after prolapse surgery reported to be as high as 30% [5].

There is recognition of the paucity of information regarding POP risk factors [6–7]. However, the established factors include advanced age, multiparity, history of hysterectomy, obesity, mode of delivery, race and chronic lung disease. Although, several of the risk factors are probably similar in both high and low-income countries, it is possible that low-resource regions might have some additional unique factors.

Studies on the prevalence of anatomical POP are rare in the Democratic Republic of Congo (DRC) but available data suggest that POP is a very common gynecological condition, reaching frequencies of more than 15% [8].

The east of DRC is an area that has been facing insecurities for decades, which has made access to quality obstetric care quite challenging. Moreover, the focus on the devastating effects of urogenital fistulae in eastern DRC and other low-income countries has resulted in very few POP related epidemiological studies despite the prevalence of this problem. Indeed, in 2021 out of the 2,507 gynecological admissions in Panzi Hospital, 425 (17%) were due to POP.

The main aim of this study was to determine risk factors for significant degree (≥ stage II) of anatomical POPs in eastern DRC (South Kivu) to inform future POP prevention policies in the region.

## Methods

This was an unpaired case-control study that took place between January 2021 and January 2022 in the gynecology department of the Panzi General Hospital in the city of Bukavu in the eastern Democratic Republic of Congo. The study was approved by the National Ethics Committee of the School of Public Health of the University of Kinshasa on 30/01/2021 (reference number: ESP/CE/20/2021).

### Study participants

Study participants were recruited from the cohort of patients attending our unit with different gynecological problems during the study period. Only those who agreed to participate and signed a written consent were enrolled into the study. Patients known to be pregnant, have a gynecological cancer and those who could not provide answers for all the variables of interest were excluded from the study.

The initial assessment, including the collection of information about the variables of interest, were performed by 4th year resident physicians. Following this, participants were examined by one of 2 experienced gynecologists. Patients were asked to have an empty bladder prior to examination, which was performed both at resting and on Valsalva using a Simm’s speculum. Participants with stage II – IV POP according to POP-Q were considered cases, while those classified as POP-Q 0 - I were considered controls. Recruitment continued till the required sample was achieved for each of the groups.

The sample size for the unmatched case-control study design was calculated using Epi Info version 7. We used family history of prolapse as a surrogate for prolapse risk factors. The frequency of this risk factor in patients diagnosed with prolapse in a urogynecology clinic was reported to be 50.8% [3]. We assumed that the frequency of the same variable in controls would be 40%. Based on a power of 80%, a 95% confidence interval, a case-control ratio of 1.0 and an odds ratio representing the association between exposure (family history of prolapse) and outcome (POP) of 1.55; we estimated that a total of 434 patients (217 cases and 217 controls) would be required.

### Study variables

The study variables of interest were age, height < 150 cm, BMI (< 18.5 kg/m2 and > 30 kg/m2), vaginal parity, menopausal status, occupation and patient reported previous obstetric history of operative vaginal delivery (OVD), use of fundal pressure (Kristeller maneuver), macrosomia (≥ 4000 g), history of previous episiotomy, spontaneous perineal tear and place of birth (home or medical facility). We also collected information about medical history of chronic constipation, chronic lung disease, diabetes and any family history of POP. Of note, even if babies are born at home, babies tend to be taken to a healthcare facility straightaway to get their exact birthweight assessed and documented.

We collected data on occupation because we were initially interested in exploring jobs associated with heavy lifting (≥ 30 kg per day). However, given that the majority of women in the study region are farmers and given the difficulty in objectively and accurately assessing the average weight of the loads carried daily, we did not include the variable ‘’carrying heavy loads’’ in the logistic regression model.

Home birth was considered a variable of interest if the patient reported at least 2 births that took place at home without medical assistance. Vaginal parity was categorized to low parity (1–2 births), multiparous (3–4 births) and grand multiparous (≥ 5 births).

Constipation was considered a variable of interest if the patient reported difficulty passing stools accompanied by hard stools for at least 3 months and chronic lung disease if they reported a persistent chronic cough for ≥ 3 months. Variables leading to increase abdominal pressure together with diabetes were analyzed together under the “medical history” variable. Family history of pelvic prolapse was considered positive if affecting a first degree female relative.

### Statistical analysis

Data were entered using Epidata 3.5 then exported to SPSS 23 software for treatment and analysis. Quantitative variables are presented as means and standard deviations, while categorical variables are expressed as absolute and relative frequencies. The Pearson Chi square test was used to test association of covariates with the outcome. The Student T test was used to compare quantitative values between outcome categories. Binary and multivariate logistic regressions were used, and crude and adjusted odds ratios (OR) were reported with 95% confidence intervals (CI). A factor was considered for multivariate model when its crude association had a p value < 0.05. To check co-linearity between risk factors, the variance inflation factor (VIF) was used. A p value < 0.05 was considered statistically significant.

## Results

The sociodemographics of the study population are presented in Table 1. There were no significant differences between cases and controls with regards to age and parity, however, the mean height and BMI were higher in the control group and this difference was statistically significant (*p* < 0.001). When comparing the clinical parameters and the variables of interest between both groups, there were statistically significant differences between cases and the controls with regards to occupation, BMI, history of episiotomy, perineal tears, place of birth and family history of prolapse (*p* < 0.001). The differences were also significant between both groups in relation to history of use of Kristeller maneuver (*p* < 0.002) and macrosomia (p 0.019) (Table 1).

The Binary and multivariate logistic regression analyses are presented in Table 2. On Binary logistic regression, out of all the *a priori* set variables of interest, BMI, home birth, family history of POP, history of birth without episiotomies, height ≤ 150 cm and a previous obstetric history of perineal tear, birth of a macrosomic baby, or the use of Kristeller maneuver during birth were significantly associated with POP. However, on multivariate analysis, only low BMI (< 18.5) (aOR 2.991 [1.419–6.307]), a height ≤ 150 cm (aOR 5.328 [2.942–9.648]), previous home birth (aOR 6.102 [ 3.526–10.561]), previous birth without episiotomy (aOR 3.504 [2.031–6.048]), delivery of a macrosomic baby (aOR 1.929 [ 1.121–3.321]) and a family history of POP (aOR 2.085 [ 1.107–3.924]) continued to be significant variables.

## Discussion

The study aimed to determine the risk factors associated with pelvic organ prolapse. The analysis showed that POP was associated with low BMI, having given birth at least twice at home, having a family history of prolapse, previous birth without an episiotomy, a height ≤ 150 cm and vaginal birth of a macrosomic baby. The risk factors for POP in low-resource settings are poorly understood and may be different from those in high-resource settings given the presence of unique sociodemographic factors, obstetrical practices and, potentially, differences in genetic predisposition. Previous studies that have attempted to address this in these settings were cross-sectional descriptive studies [4–9–10–11–12].

In this study, we compared patients presenting with stage II-IV POP to those presenting for routine gynecological care with no evidence of clinically significant prolapse. We included patients with stage II POP because, even at this stage, patients have tendency to report significant functional impairments [13].

Contrary to several studies [13–15], age and parity were not identified as factors associated with POP in our population. The average parity in both of our study cohorts was 7 ± 3 with no statistically significant difference between cases and the controls. This prompts us to question whether the number of vaginal births or the events related to these births that has an impact on the pelvic floor? Parity considered and reported in our study relates to the number of vaginal births only, and hence, vaginal parity. It has been demonstrated that the distention of the vaginal canal by the descending fetus results in stretch of the levator ani muscles and pudendal nerves resulting in neuropathy, muscle wasting and POP [16–17]. Our data suggest that the events associated with these vaginal births (i.e. quality) rather than their number (i.e. quantity) seems to be a more important factor. Indeed, in this study, home birth, delivering a macrosomic baby and history of birth without an episiotomy were significantly associated with POP. Furthermore history of perineal tear and the use of Kristeller maneuver were significantly associated with POP in our bivariate analysis. Nevertheless, this association did not persist on multivariate analysis.

Swift at al [13] suggested that there was a 24% increase in the incidence of severe POP with every 1 lb increase in the weight of the largest baby delivered vaginally. Additionally, Snooks and associates noted a relationship between increased birthweight and damage to the pudendal nerve at the time of childbirth [16]. Prolonged labor (likely to happen in unattended home births), episiotomies, tears of the perineum and birth of a macrosomic baby are conditions that have been associated with levator ani muscle avulsion [18–19 –20]. Although only speculative, it is possible that this is the mechanism by which the risk factors identified in our study would have been associated with POP. This reinforces the concept that it is the perinatal events that play the most important role in the development of POP rather than the number of deliveries.

Epidemiological studies performed on Caucasian populations have identified age and menopause as major factors in the development of pelvic floor disorders [21]. Trowbridge et al. reported a decline in urethral sphincter function with increasing age independent of vaginal deliveries [22]. This age-related muscle deterioration, likely, contributes to pelvic floor problems that develop years after childbirth. However, age was not identified as a significant variable in any of our analyses. Interestingly, epidemiological studies [4 -9- 12] carried out in low-resource settings and on black populations have noted that POP tends to occur at a younger age in these settings. The much higher life expectancy in higher income settings could have confounded these findings.

We identified low BMI (BMI < 18.5) as a risk factor for POP (OR 2.991; 95% CI 1.419–6.307; p 0.004). Being overweight or obese were not factors associated with POP in our study. Interestingly, our findings are not consistent with most research findings, which have demonstrated the association between high BMI and POP. A recent systematic review and meta-analysis showed that obese and overweight women were more likely to develop POP compared to women with a normal BMI [23]. Nevertheless, it is important to note that only one of the 22 eligible studies in the meta-analyses was conducted in an African context. Most of the studies were conducted in Europe and America where the prevalence of high BMI tends to be higher. In our study region, most obese and overweight women come from affluent backgrounds and are therefore less exposed to suboptimal obstetrical conditions such as home birth and to other risk factors such as carrying heavy loads. It would be interesting to explore the relationship between nutrition and pelvic floor health further in specifically designed epidemiological studies.

Family history was identified as a non-modifiable potential risk factor for POP suggesting a potential genetic link (OR 2.085; 95% CI 1.107–3.924). A genetic predisposition to the development of prolapse has long been suspected and provides a plausible explanation to the increased risk of POP in women with a positive family history [20]. In 2006 Jack et al. performed genetic analysis on 10 families of women with advanced POP and noted a link [24]. Advances in genome mapping have made it possible to study specific genes and identify their contributions to the development of selective diseases. In 2015, a study from Utah described genes on chromosomes 10 and 17 that predisposed to POP [25]. These results support the role that genetics might play in predisposing some women to the development of POP and other pelvic floor disorders.

We also found a significant association between a height of ≤ 150 cm and POP on multivariate analysis (aOR 5.328; 95% CI 2.942–9.648; *p* < 0.001). Stature is associated with skeletal dimensions including the of the pelvis. The size of the area of pelvic outlet covered by the pelvic floor is also of mechanical importance. It has been suggested that the larger the size of the outlet, the greater the force applied on pelvic structures, which could play a role in the development of prolapse [26]. Furthermore, variations in pelvic dimensions are known to have important perinatal implications, which can affect pelvic floor function. In view of this complex relationship, the relationship between height and POP should be interpreted with caution and warrants further investigation.

### Limitations and strengths of the study

We recognize that our study has several limitations. It is possible that participants could have under or over-reported certain variables, particularly those related to their previous obstetric history which could have introduced recall bias into our findings. We were also not able to objectively and accurately assess the average weight each participant carried per day during their normal daily activities. We believe that this is an important risk factor, especially in the study setting, which may constitute a potential source for information bias into our series. To mitigate the risk of introducing any inaccuracies into our results, we opted not to include this variable in our regression analyses. Furthermore, there are other potential risk factors that are particular to our study population, like short inter-pregnancy interval, however, accurate information about this variable was not readily available for us to include. Nonetheless, the fact that it is one of the few case-control studies reporting potential risk factors for POP at east of Democratic Republic of Congo, the use of a standardized and validated tool to categorize the study cohort and the statistical methodology to eliminate confounding variables are all major strengths to our work.

## Conclusion

A significant proportion of patients in the study area have pelvic organ prolapse. There are several demographic and obstetric variables, including low BMI, a height equal to or less than 150 cm, home birth, family history of prolapse, history of birth without an episiotomy and previous delivery of a macrosomic baby, that were significantly associated with prolapse in our study cohort. Although our results are preliminary, they provide the initial steps towards understanding risk factors for developing pelvic organ prolapse in low-resource settings. Findings from this study are the initial steps to the possibility of developing personalized risk prediction models that can help patients and healthcare professionals make informed decisions about intrapartum care to mitigate the risk of POP. Finally, our study supports the view that effective pelvic floor disorder prevention models should be multifaceted to tackle the multiple demographic, environmental, genetic and birth related factors that predispose to these conditions.

**Table 1:** Distribution of sociodemographic and clinical variables between cases (Prolapse) and controls (No prolapse)

Variables	No prolapse (Controls)	Prolapse (Cases)	P-value
	Mean (SD) or n (%)		
Age	46.4 ± 11.92	46.65 ± 14.08	0.843
Parity	7 ± 3	7 ± 3	0.042
Height	160 ± 9	152 ± 7	< 0.001
BMI	23.84 ± 3.79	20.68 ± 3.57	< 0.001
Underweight (BMI < 18.5)	4 (0.90)	52(12.00)	< 0.001
Normal (BMI (18.5–24.9)	160 (36.90)	143(32.90)	
Overweight (BMI 25–29.9)	38 (8.80)	7 (1.60)	
Obese (BMI ≥ 30)	15 (3.50)	15 (3.50)	
Occupation			
Household	95 (43.78)	11 (5.07)	< 0.001
Other occupations	49 (22.58)	12 (5.53)	
Farmer	73 (33.64)	194 (89.40)	
Place of delivery			
Home	37 (17.05)	128 (58.99)	< 0.001
Maternity	180 (82.95)	89 (41.01)	
Family history of prolapse	38 (8.80)	68(15.70)	0.001
Previous episiotomy	141 (37.20)	54 (14.20)	< 0.001
Menopause	97 (22.40)	106 (24.40)	0.387
Height			
Height ≤ 150 cm	29 (6.70)	94 (21.70)	< 0.001
Height > 150 cm	188 (43.30)	12 3(28.30)	
History of perineal tear	**13 (3.00)**	**40 (9.20)**	< 0.001
History of macrosomia (≥ 4000 g)	**77 (17.70)**	**101 (23.30)**	0.019
History of OVD	**12 (2.80)**	**17 (3.90)**	0.336
Kristeller maneuver			
No	122 (28.10)	89 (20.50)	0.002
Yes	95 (21.90)	128 (29.50)	
Medical disorders	76 (17.50)	68 (15.70)	0.415
Age			
18–30 years old	27 (6.20)	35 (8.10)	0.081
31–49 years old	108 (24.90)	85 (19.60)	
50 years and over	82 (18.90)	97 (22.40)	
Vaginal parity			
≤ 2	22 (5.10)	19 (4.40)	0.646
3–4	50 (11.50)	44 (10.10)	
≥ 5	145 (33.40)	154 (35.50)	

**Table 2:** Binary and multivariate Logistic Regression analyses

Variables	Binary logistic regressIon			MultivariAt Logistic regression		
	**OR**	**95% CIfor EXP(B)**	**p**	**aOR**	**95% CI**	**p**
Low BMI	2.864	1.671–4.909	< 0.000	2.991	1.419–6.307	0.004
Home birth	6.997	4.482–10.922	< 0.001	6.102	3.526–10.561	< 0.001
Family history of POP	2.15	1.367–3.380	0.001	2.085	1.107–3.924	0.023
Previous birth without episiotomy	5.015	3.239–7.765	< 0.001	3.504	2.031–6.048	< 0.001
Pre-menopausal	1.181	0.81–1.723	0.387	-	-	-
Height ≤ 1.50 m	4.954	3.083–7.961	< 0.001	5.328	2.942–9.648	< 0.001
History of perineal tear	3.546	1.838–6.843	< 0.001	-	-	-
History of macrosomia (≥ 4000 g)	1.583	1.077–2.327	0.019	1.929	1.121–3.321	0.018
History of OVD	1.452	0.676–3.118	0.339	-	-	-
History of Kristellar maneuver	1.847	1.262–2.703	0.002	-	-	-
No medcial history*	1.181	0.792–1.762	0.415	-	-	-
18–30 years old	1.096	0.612–1.961	0.758	-	-	-
31–49 years old	0.665	0.442–1.001	0.051	-	-	-
Multiparity	1.019	0.488–2.126	0.96	-	-	-
Grand multiparity	1.23	0.639–2.366	0.536	-	-	-

*No medical history of chronic constipation, chronic lung disease or diabetes, OR : odds ratio, aOR : Adjusted odds ratio

## Data Availability

The datasets used and/or analyzed during the current study are available from the corresponding author on reasonable request.

## Abbreviations

aOR: Adjusted Odds Ratios
OR: Odds Ratios
OVD: Operative vaginal delivery
POP: Pelvic organ prolapse
DRC: Democratic Republic of Congo
